# Therapeutic Potential of Triptolide as an Anti-Inflammatory Agent in Dextran Sulfate Sodium-Induced Murine Experimental Colitis

**DOI:** 10.3389/fimmu.2020.592084

**Published:** 2020-11-09

**Authors:** Bufu Tang, Jinyu Zhu, Baohui Zhang, Fazong Wu, Yajie Wang, Qiaoyou Weng, Shiji Fang, Liyun Zheng, Yang Yang, Rongfang Qiu, Minjiang Chen, Min Xu, Zhongwei Zhao, Jiansong Ji

**Affiliations:** ^1^ Key Laboratory of Imaging Diagnosis and Minimally Invasive Intervention Research, Lishui Hospital, School of Medicine, Zhejiang University, Lishui, China; ^2^ Department of Radiology, Second Affiliated Hospital, School of Medicine, Zhejiang University, Hangzhou, China; ^3^ Department of Physiology, School of Life Science, China Medical University, Shenyang, China; ^4^ Department of Radiology, The Fifth Affiliated Hospital of Wenzhou Medical University, Lishui, China

**Keywords:** colitis, PDE4B, reactive oxygen species, macrophage, triptolide

## Abstract

Inflammatory bowel disease (IBD), which includes ulcerative colitis (UC) and Crohn’s disease (CD), is a group of chronic and incurable inflammatory diseases involving the gastrointestinal tract. In this study, we investigated the anti-inflammatory effects of triptolide in a dextran sulfate sodium (DSS)-induced mouse colitis model and LPS-activated macrophages and explored the specific molecular mechanism(s). In mice, triptolide treatment showed significant relief and protection against colitis, and it markedly reduced the inflammatory responses of human monocytes and mouse macrophages. Pharmacological analysis and weighted gene co-expression network analysis (WGCNA) suggested that PDE4B may be an important potential targeting molecule for IBD. Exploration of the specific mechanism of action indicated that triptolide reduced the production of ROS, inhibited macrophage infiltration and M1-type polarization by activating the NRF2/HO-1 signaling pathway, and inhibited the PDE4B/AKT/NF-*κ*B signaling cascade, which may help weaken the intestinal inflammatory response. Our findings laid a theoretical foundation for triptolide as a treatment for IBD and revealed PDE4B as a target molecule, thus providing new ideas for the treatment of IBD.

## Introduction

Inflammatory bowel disease (IBD), which includes ulcerative colitis (UC) and Crohn’s disease (CD), is a group of complex chronic inflammatory diseases that mainly affect the ileum, rectum, and colon (1). The incidence of IBD is increasing yearly worldwide (2), and interestingly, clinical studies have found that patients with IBD usually have a higher risk of colon cancer than those without IBD (3). The clinical symptoms of IBD patients are mainly manifested as recurrent diarrhea, abdominal pain, weight loss, and mucopurulent bloody stool (4). Currently, it is generally believed that the pathogenesis of IBD is closely related to innate immunity, environment, reactive oxygen species (ROS), and other factors (5), but the specific pathogenesis is not clear. Moreover, due to the complex condition, long course, frequent recurrence, and difficult treatment of IBD, it is particularly important to explore new targets for IBD and seek new effective therapeutic drugs for IBD.

The inflammatory response is a complex physiological process and plays an important role in inflammation-related diseases, including enteritis (6), cardiovascular diseases (7), obesity, and cancers (8). Macrophages are phagocytic cells derived from circulating monocytes and are present in all mammalian tissue types. Macrophages are regarded as important responders and regulators of many physiological systems, including development, homeostasis, repair, immune responses, and defense against pathogens (9, 10). Changes in external stimuli and conditions induce macrophage polarization to different phenotypes, including the M1 phenotype (proinflammatory) and M2 phenotype (anti-inflammatory).

Macrophages are key participants in IBD. M1 macrophages and proinflammatory cytokines aggravate IBD, while M2 macrophages can promote tissue repair and release of anti-inflammatory cytokines to reduce IBD symptoms (11, 12). Previous studies have demonstrated that the number of M1 macrophages increases and that of M2 macrophages decreases in colitis, accompanied by the induction of inflammatory cytokines and the inhibition of anti-inflammatory cytokines (13, 14). In addition, the involvement of ROS has also been suggested in the development of IBD (15). Endogenous ROS are mainly produced in organelles such as the endoplasmic reticulum, mitochondria, and peroxisomes, and the mitochondrial electron transport chain is responsible for the majority of ROS production (16). Excessive ROS production leads to reduced ATP production, inhibition of intracellular electron transport chains, and mitochondrial DNA damage, which in turn leads to cell death (17). CD has been reported to be associated with an oxidative stress imbalance including increased ROS and decreased net antioxidant activity (18). During the intestinal inflammation of IBD, the oxidative stress generated by ROS stimulates the initial inflammatory response through positive feedback and leads to additional ROS production and further tissue damage (19).

Dextran sulfate sodium (DSS) is a chemical inflammatory agent which, when added to the drinking water of mice, can induce IBD (20). In adult mice, DSS supplementation of the drinking water can be utilized to construct experimental IBD models (21). The clinical phenotype of this model is highly similar to that of human UC and includes bloody stools, diarrhea, and weight loss. In addition, this model can induce the typical pathological characteristics of colitis, such as mucosal barrier damage and immune cell infiltration (22, 23). Therefore, considering the widespread use of DSS to create an inflammatory animal model, we hoped to explore the effect of triptolide on IBD by constructing a DSS-induced mouse colitis model.

Triptolide, a diterpenoid extract of *Tripterygium wilfordii* Hook F, has a wide range of pharmacological activities, including antitumor, antifertility, and anti-inflammatory activities (24, 25). However, it has been difficult to mechanistically determine whether triptolide treatment regulates the DSS-induced intestinal inflammation and macrophage homeostasis *in vivo* and *in vitro*. Lipopolysaccharide (LPS) is the principal component of the outer membrane of gram-negative bacteria and is considered an important proinflammatory agent (26). Stimulation with LPS and IFN*γ* triggers macrophage polarization to a proimmune (M1-like) phenotype, resulting in the release of large quantities of proinflammatory mediators, including interleukin (IL)-1β, IL-6, tumor necrosis factor-α (TNF-α), and ROS (27, 28). In recent years, studies have attempted to determine the mechanism involved in the regulation of the intestinal inflammatory response, but the mechanism is still unclear.

In this study, we constructed a DSS-induced mouse colitis model to explore the protective effect and molecular mechanism of action of triptolide on DSS-induced colitis. Subsequently, we further analyzed the regulatory effects of triptolide on macrophage infiltration and polarization and secretion of inflammation-related factors in DSS-induced mouse colitis models and LPS-activated RAW264.7 cells. Pharmacology analysis and weighted gene coexpression network analysis (WGCNA) were performed to further explore the potential mechanism of triptolide and the potential therapeutic target in IBD. This study demonstrates for the first time that triptolide treatment protects against colitis by interfering with infiltration and polarization of macrophages and secretion of inflammation-related factors, clarifies specific molecular mechanisms, and explores potential therapeutic targets. Our study provides new ideas for the treatment strategy of IBD.

## Materials and Methods

### Acquisition of Differentially Expressed Genes in IBD

The mRNA sequencing data and corresponding clinical information of IBD patients were obtained from the GEO dataset (GSE75214) that includes 97 UC samples, 8 CD samples, and 11 normal samples. The limma R software package was utilized to determine DEGs with absolute log 2-fold change (FC) >1 and adjusted P value <0.05.

### Weighted Gene Co-Expression Network Analysis

WGCNA (29) was used to construct a gene co-expression network of DEGs between IBD samples and normal samples in the GEO dataset (GSE75214), thereby identifying co-expression gene modules that are highly relevant to UC and CD and determining the core genes closely related to the progression of UC and CD. The expression characteristics of DEGs were adopted as the input of WGCNA, and UC and CD were defined as the phenotype of the samples. The soft threshold parameter was set as *β* = 8 and scale-free R 2 = 0.80 to ensure a signed scale-free co-expression gene network. Using an expression matrix and *β* value to construct a co-expression matrix, genes with similar expression patterns were classified into the same gene module to produce co-expression modules. The Eigengenes function was used to calculate the differences in the module eigengenes (MEs) and the correlation of the module with UC and CD. A heatmap was generated to visualize the correlation of each module with UC and CD. The Pearson coefficient was calculated to evaluate the correlation between the genes in the module and UC and CD and determine the core genes. The above analysis was achieved using the R package “WGCNA”.

### Determination of Immune Cell Infiltration

CIBERSORT analysis (30) was applied to quantitatively convert the transcriptome data in the GSE75214 dataset into the absolute abundance of ten types of immune and stromal cells in heterogeneous tissues and then to evaluate the infiltration level of 22 human immune cell subsets in the heterogeneous tissues. The R package “CIBERSORT” was used to convert the mRNA data to the infiltration level of immune cells in the tissue microenvironment.

### Induction of Colitis in Mice and Treatment

Male C57BL/6 wild-type mice were obtained from Beijing Vital River Laboratory Animal Technology (Beijing, China). DSS was purchased from MP Biomedicals (Costa Mesa, CA, USA) and triptolide from MedChemExpress (Shanghai, China). Mice were randomly assigned to three groups, namely, the control group, colitis group and treatment group (n = 6). The control group was provided drinking water without DSS, while the colitis group and the treatment group were given water containing 2.5% DSS; the mice in the treatment group were intraperitoneally injected with 0.02 mg/kg triptolide every other day. The animals’ conditions were monitored through general inspections and weight change. On the fifth day, the colitis group and the treatment group were switched to drinking water without DSS. On the eighth day, the mice were euthanized by neck dislocation under isoflurane anesthesia, and the entire colon was removed, rinsed gently with PBS, and dried on filter paper to measure its length. The colon was then divided into sections for immunological and western blot analysis.

### Animals and Macrophage Extraction

Male C57BL/6 wild-type mice were treated with 2.5% DSS to establish the colitis mouse model and were then treated with 0.2 mg/kg triptolide (I.P, once every two days) in DSS-induced colitis. Mouse peritoneal macrophages were induced in C57BL/6 mice by intraperitoneal injection of 2.5 ml of sterile 4% Brewer thioglycollate. Cells were harvested 2–3 days later by peritoneal lavage and cultured on plates. After 24 h at 37°C in a constant temperature incubator, non-adherent cells were removed using cold phosphate-buffered saline (PBS), and adherent macrophages were used for further studies.

### Cell Cultures and LPS and Triptolide Treatments

The murine RAW264.7 macrophage and human U937 monocyte cell lines were obtained from the American Type Culture Collection (Manassas, VA, USA), and ML385 was purchased from MedChemExpress (Shanghai, China). Peritoneal macrophages, RAW264.7, and U937 cells were cultured in RPMI 1640 medium supplemented with 2 mM L-glutamine, 100 U/ml penicillin–streptomycin, and 10% fetal bovine serum in 5% CO_2_ at 37°C in a constant temperature incubator. The cells were treated with 1,000 ng/ml LPS plus 0–40 nM of triptolide and 2 uM ML385 for 24 h and then used for further experiments.

### RNA Preparation and the Quantitative Real-Time Polymerase Chain Reaction

Total RNA was extracted using TRIzol^®^ reagent, and the cDNA was prepared using the TransScript Top Green qPCR supermix (TransGen, Guangzhou, China) according to the manufacturer’s instructions. Relative gene expression was assessed with a RT-PCR detection system (Bio-Rad Laboratories, Hercules, CA, USA) utilizing Real-Time PCR Master Mixes (TransGen) according to the manufacturer’s instructions. Sequences of primers used for RT-PCR are shown in **Table S1**.

### Western Blotting

Lysates of total protein were obtained using radioimmunoprecipitation assay buffer (Beyotime Biotechnology, Shanghai, China), and the lysates were incubated in ice before the supernatants were collected. A wet western blotting system was used with *β*-actin antibody as the internal control (Cell Signaling Technology, Beverly, MA, USA). A and B luminescent solutions of ECL hypersensitive chemical luminescence reagents were diluted in equal proportions and dripped onto the blot, and the blot was imaged with an Amersham Sensor 600 (GE Medical, Chicago, USA). Details of the antibodies used for western blotting are listed in **Table S2**.

### Isolation of Macrophages in Colon Tissue

EDTA, penicillin, and streptomycin were obtained from MedChemExpress (Shanghai, China). Collagenase IV and DNase I were purchased from Thermo Fisher Scientific (Shanghai, China). The colons of the mice were excised and opened longitudinally, the intestinal contents were removed, and the intestinal tissues were washed with PBS, and then the intestinal tissues were cut into 5-mm pieces. The intestinal tissue samples were gently shaken at 250 rpm for 30 min at 37°C in PBS containing 10% (vol/vol) FBS, 5 mM EDTA, 100 units/ml penicillin and 100 μg/ml streptomycin to remove colonic epithelial cells (31). The tissues were washed extensively in PBS several times until the supernatant became transparent to further remove residual epithelial cells. Then the tissues were further cut into smaller pieces and placed in PBS containing 10% (vol/vol) FBS, 300 U/ml collagenase IV, and 5 U/ml DNase I, 100 units/ml penicillin, and 100 μg/ml streptomycin for 40 min with gentle shaking at 250 rpm at 37°C. The product was filtered through a 70-μm cell filter and washed twice with PBS to obtain a single cell suspension. After blocking the Fc receptor, the cells were labeled with biotin-conjugated anti-CD11b antibody (BioLegend) and then incubated with anti-biotin microbeads (Miltenyi Biotec) (32). LS columns (Miltenyi Biotec) were used for magnetic-activated cell sorting on a MidiMACS Separator (Miltenyi Biotec).

### Flow Cytometry to Measure Macrophage Polarization

Flow cytometry was used to measure the phenotypical changes in M1-mediated markers in RAW 264.7 macrophages. CD80 (Becton Dickinson, Franklin Lakes, NJ, USA) was regarded as the specific surface markers of the M1 phenotype of macrophages. The antibodies used for the fluorescence-activated cell sorting (FACS) are shown in **Table S2**. And the quantitative analysis of macrophage polarization was used by Flow Jo software.

### Measurement and Quantification of ROS Levels

The intracellular ROS levels of intestinal macrophages in mouse colitis tissues and RAW264.7 macrophages were detected by using the DCFH-DA probe (Yeasen, Shanghai, China). Intestinal macrophages were cultured in basic RPMI 1640 medium in the humidified cell incubator with an atmosphere of 5% CO2 at 37°C for 4 h. Then 0.1% DCFH-DA was added to the cells and incubated for 20–30 min, then washed with cold PBS 2–3 times to measure intracellular ROS levels. RAW264.7 macrophages were incubated in basic RPMI 1640 medium with 1,000 ng/ml LPS in the humidified cell incubator with an atmosphere of 5% CO2 at 37°C for 24 h and treated with or without triptolide and ML385 at the indicated concentrations for another 24 h. Then 0.1% DCFH-DA was added to the cells and incubated for 20–30 min, and the cells were washed with cold PBS 2–3 times. Intracellular ROS levels were detected by flow cytometry or fluorescence microscopy according to the manufacturer’s instructions. The quantitative analysis of intracellular ROS levels were used Flow Jo software (https://www.flowjo.com/index.php) or ImageJ software (https://imagej.nih.gov/ij/).

### Immunofluorescence Assay

An immunofluorescence assay was used to detect nuclear and cytoplasmic Nrf2, HO-1, phosphorylated P65, and PDE4B expression levels. DAPI (4′,6-diamidino-2-phenylindole) was utilized as a nuclear marker, and all samples were analyzed using an FV10-ASW instrument (Olympus, Tokyo, Japan) and the installed software.

### Statistical Analysis

Statistical analyses were performed using SPSS statistical software for Windows, version 16.0 (SPSS, Chicago, IL, USA). Values were expressed as the mean ± SD. Quantitative data in paired groups were determined using the Student’s *t*-test. One-way analysis of variance was performed for multiple group comparisons. A value of P < 0.05 indicated a significant difference.

## Results

### Triptolide Attenuated DSS-Induced Intestinal Inflammation in the Mice

**Figure 1:**
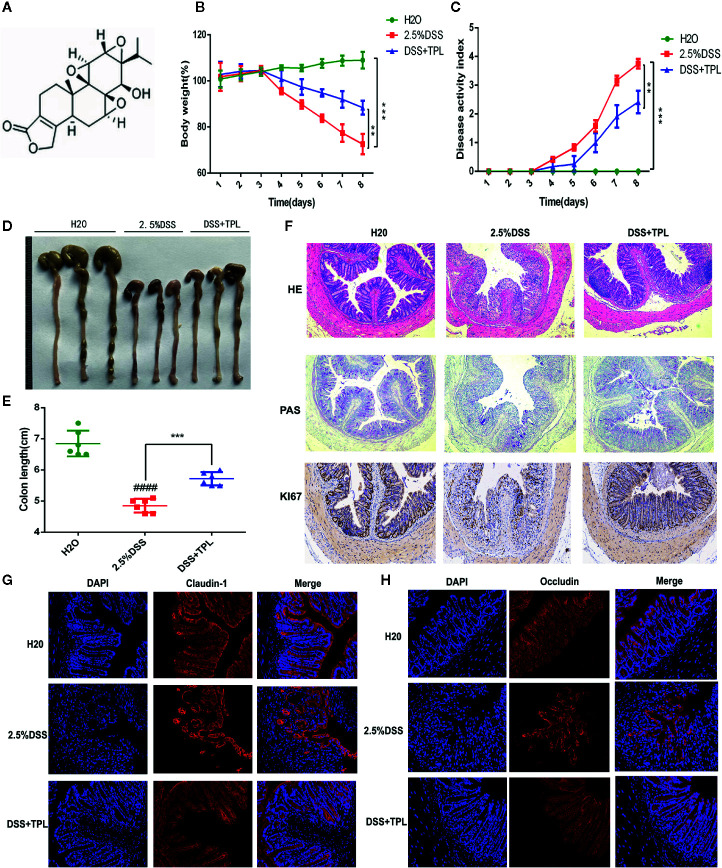
The protective effect of triptolide on DSS-induced colitis mice. **(A)** The chemical structure of triptolide is shown. Mice aged 6–8 weeks were treated with 2.5% DSS with or without triptolide administration. **(B)** The body weight and **(C)** disease activity index (DAI) of these mice during the experimental period are depicted. **(D–F)** The pathological features of colitis are depicted by HE staining, PAS staining, and immunohistochemical assay of Ki67 levels. The expression of **(G)** claudin-1 and **(H)** occludin are indicated by immunofluorescence assay. Data are shown as the mean ± SD of at least three independent experiments. **P < 0.01; ***P < 0.001. ^####^ means that the statistical difference between the results of the 2.5% DSS group and the H2O group was P < 0.0001.

First, a flowchart of this study was constructed to intuitively describe our research process (**Figure S1**). To explore the anti-colitis effect of triptolide, the mice were administered 2.5% DSS to establish the colitis mice model and were then treated with 0.2 mg/kg triptolide. The DSS-induced murine experimental colitis model with or without triptolide administration is shown in **Figure S2**. The chemical structure of triptolide is shown in **Figure 1A**. Clinical symptoms and pathological features were used to evaluate the protective effect of triptolide in DSS-induced colitis. Triptolide treatment obviously alleviated body weight loss and the disease activity index (DAI) caused by DSS treatment (**Figures 1B, C**). DAI is a common index used to assess the severity of colitis combined with body weight loss, stool consistency and the presence of blood in the stool. In addition, triptolide significantly alleviated the colon shortening effect of DSS treatment (**Figures 1D, E**). These findings suggest that triptolide treatment effectively alleviated the clinical symptoms in DSS-induced colitis. To further evaluate the anti-inflammatory role of triptolide, HE staining, PAS staining, and immunofluorescence was used to assess pathological features in DSS-induced colitis. Triptolide treatment significantly attenuated the colon tissue injury caused by DSS treatment. Moreover, the levels of Ki67 substantially increased in DSS-induced colitis tissue, while triptolide treatment significantly reduced the expression of Ki67 (**Figure 1F**). In a previous study, the expression of claudin-1 and occludin, important indicators of the integrity of the intestinal mucosal barrier, were inhibited in DSS-induced colitis. Immunofluorescence assay showed that triptolide treatment significantly upregulated the level of claudin-1 and occludin in DSS-induced colitis (**Figures 1G, H**). Taken together, these results indicated that triptolide treatment attenuated the DSS-induced intestinal inflammatory response in the mice.

### Triptolide Inhibited Macrophage Infiltration and Inflammatory Cytokines *In Vivo* and *In Vitro*

**Figure 2:**
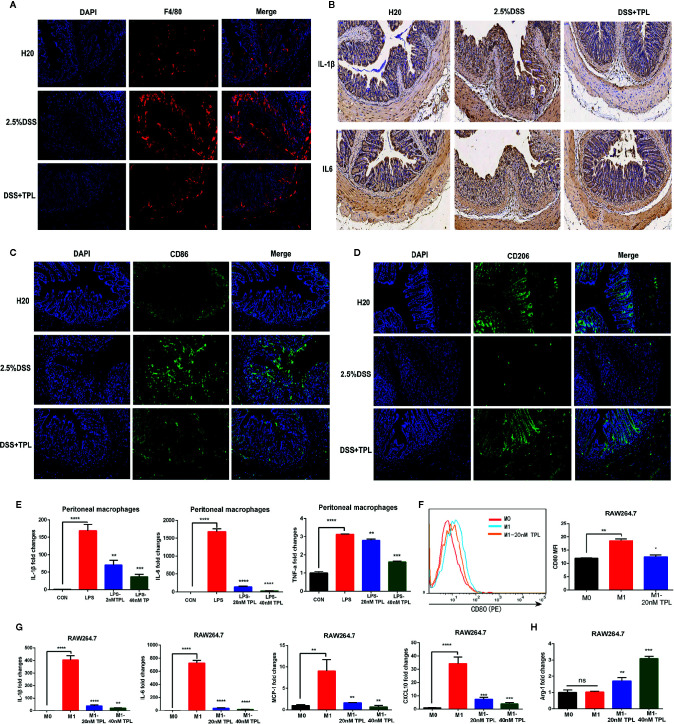
Triptolide inhibited macrophage infiltration and inflammatory cytokines *in vivo* and *in vitro*. Mice aged 6–8 weeks were treated with 2.5% DSS with or without triptolide administration. **(A)** The F4/80 expression was measured by immunofluorescence assay. **(B)** The IL-1β and IL-6 level of colitis was detected by immunohistochemistry assay. RAW 264.7 macrophages were treated with 1,000 ng/ml lipopolysaccharide (LPS) with 0–40 nM triptolide for 24 h. **(C)** The CD86 expression was measured by immunofluorescence assay. **(D)** The CD206 expression was measured by immunofluorescence assay. **(E)** RT-qPCR was used to measure the levels of proinflammatory cytokines such as IL-1β, IL-6, and TNF-α. RAW 264.7 macrophages were treated with 500 ng/ml LPS and 100 ng/ml IFN*γ*, then incubated with 0–40 nM triptolide for 24 h. **(F)** Flow cytometry was performed to determine the expression of CD80, a M1-mediated surface marker. **(G)** RT-qPCR was used to measure the levels of M1-mediated cytokines such as IL-1β, IL-6, MCP-1, and CXCL10. **(H)** RT-qPCR was used to measure the levels of M2-mediated cytokines including Arg-1. *β*-actin served as an internal control. Data are shown as the mean ± SD of at least three independent experiments. *P < 0.05; **P < 0.01; ***P < 0.001; ****P < 0.0001; and ns means no significance.

Macrophages function as a primary regulator of inflammatory responses and play a central role in inflammation-associated disorders, including IBDs. To explore the potential role of triptolide in DSS-induced colitis, immunofluorescence, immunohistochemistry, and RT-qPCR were used to measure macrophage infiltration and inflammatory factors. Triptolide treatment effectively inhibited macrophage infiltration in DSS-induced colitis (**Figure 2A**). Triptolide also suppressed IL-1β and IL-6 expression in mouse colitis tissue (**Figure 2B**). To explore the effect of triptolide on the polarization of macrophages in mouse colitis tissues, we used CD86 as the marker of M1 macrophage and CD206 as the marker of M2 to detect the infiltration of M1 and M2 macrophages *in vivo* by immunofluorescence. The results showed that triptolide treatment inhibited the polarization of M1 macrophages (**Figure 2C**) and promoted the polarization of M2 macrophages (**Figure 2D**). To further characterize the role of triptolide in the LPS-stimulated inflammatory response, RAW264.7 cells were treated with 1,000 ng/ml LPS plus 0–40 nM triptolide. Our results showed that triptolide treatment significantly suppressed the proinflammatory cytokines, including IL-1β, IL-6, and TNF-α in LPS-activated macrophages (**Figure 2E**).

To confirm the role of triptolide in macrophage polarization, murine RAW 264.7 macrophages and human U937 macrophages were treated with 500 ng/ml LPS plus 200 ng/ml IFNγ with 20 nM triptolide for 24 h. Flow cytometry and RT-qPCR were used to measure the polarization of macrophages. CD80 is known to be a specific surface marker of the M1 phenotype. Our results showed that the expression levels of CD80 were inhibited in LPS-activated macrophages under triptolide treatment (**Figure 2F**), indicating that triptolide inhibited the polarization of M1 macrophages. To further determine the role of triptolide in macrophage differentiation, RT-qPCR was used to detect the inflammatory cytokines that are involved in the M1 and M2 phenotypes. Proinflammatory cytokines, including IL-1β, IL-6, MCP-1, and CXCL10, were identified as M1-mediated cytokines, and anti-inflammatory factors including ARG-1 were identified as M2-mediated cytokines. We found that triptolide treatment dramatically decreased the levels of IL-1β, IL-6, MCP-1, and CXCL10 in LPS-triggered macrophages (**Figure 2G**). Notably, triptolide treatment effectively increased the expression of ARG-1, which acted as a M2-mediated specific marker in LPS-stimulated RAW 264.7 macrophages (**Figure 2H**). In a similar manner, triptolide treatment effectively inhibited M1-mediated cytokine production, including IL-1β, IL-6, TNF-α, and CXCL10 in LPS plus IFN*γ* activated U937 macrophages (**Figure S3A–D**). Furthermore, triptolide treatment significantly upregulated the levels of M2-mediated factors, including ARG-1 and CCL22 in U937 macrophages (**Figures S3E, F)**. Together, these results indicated that triptolide treatment suppressed macrophage differentiation to the proinflammatory macrophage class and enhanced anti-inflammatory cytokine levels *in vivo* and *in vitro*.

### Triptolide Treatment Decreased ROS Generation in LPS-Activated Macrophages

**Figure 3:**
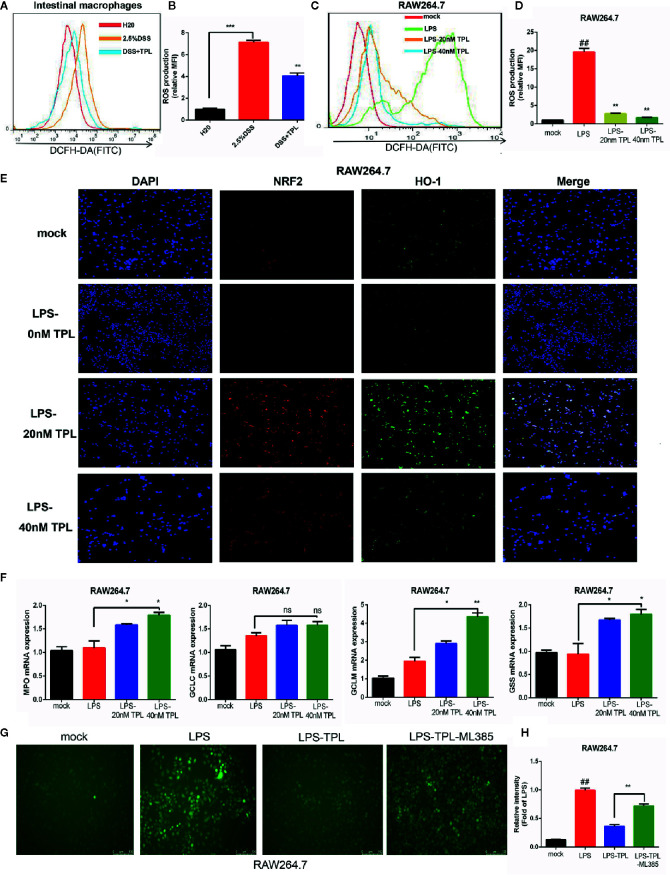
Triptolide treatment decreased the generation of reactive oxygen species (ROS) by activating NRF2/HO-1 signaling cascades. RAW 264.7 macrophages were treated with 1,000 ng/ml LPS with 0–40 nM triptolide for 24 h. **(A, B)** Flow cytometry was performed to determine the generation of ROS in intestinal macrophages in DSS-induced mice colitis. **(C, D)** The DCFH-DA probe was used to measure the ROS generation in RAW264.7 cells by flow cytometry. **(E)** Immunofluorescence assays were performed to detect the cytoplasmic and nuclear NRF2 and HO-1 levels in RAW 264.7 macrophages. **(F)** RT-qPCR was used to detect the mRNA expression of *MPO*, *Gss*, *Gclc*, and *Gclm* in RAW 264.7 macrophages. **(G, H)** The ROS inhibition effect of triptolide was significantly reversed by the ML385 in LPS-activated macrophages. *β*-actin or glyceraldehyde 3-phosphate dehydrogenase served as an internal control. Data are shown as the mean ± SD of at least three independent experiments. *P < 0.05; **P < 0.01; ***P < 0.001; and ns means no significance. ## means the statistical difference between the results of the LPS group and the mock group was P < 0.01.

During IBD intestinal inflammation, ROS are generated excessively at the inflammation site to cause further tissue damage (19). For confirming the effect of triptolide on mouse colitis, we detected the generation of ROS in intestinal macrophage in mouse enteritis tissues and found that triptolide treatment effectively reduced the release of ROS in enteritis tissues (**Figures 3A, B**). Increasing evidence has shown that in response to an LPS stimulus, induced macrophages generate large amounts of ROS in order to aggravate the inflammatory response. To further investigate the role of triptolide in the LPS-triggered inflammatory process, RAW 264.7 macrophages were treated with 1,000 ng/ml LPS with increasing amounts of triptolide for 24 h. The DCFH-DA probe was used to measure the generation of ROS. We found that with gradually increasing triptolide concentrations, LPS-triggered ROS production was dramatically inhibited (**Figures 3C, D**). To further characterize the mechanism of action, an immunofluorescence assay and RT-qPCR were used to determine the upstream or downstream genes that were associated with ROS generation. Our results showed that nuclear *NRF2* and *HO-1* were increased with increasing triptolide treatments (**Figure 3E**). Furthermore, the downstream components of the NRF2/HO-1 signaling pathway, including *MPO*, *GCLM*, and *GSS*, were also upregulated in LPS-activated macrophages. However, triptolide treatment barely changed the mRNA expression of GCLC in LPS-activated macrophages (**Figure 3F**). To determine the effect of NRF2 signaling pathway on triptolide’s inhibition of ROS production, RAW264.7 cells were incubated with the NRF2 inhibitor ML385 and triptolide, and result showed that the ROS inhibition effect of triptolide was significantly reversed by the ML385 in LPS-activated macrophages (**Figures 3G, H**). These results indicated that triptolide treatment decreased LPS-stimulated ROS generation by activating the NRF2/HO-1 signaling cascades in RAW264.7 cells.

### The Potential Therapeutic Target of Triptolide in Inflammatory Bowel Diseases

**Figure 4:**
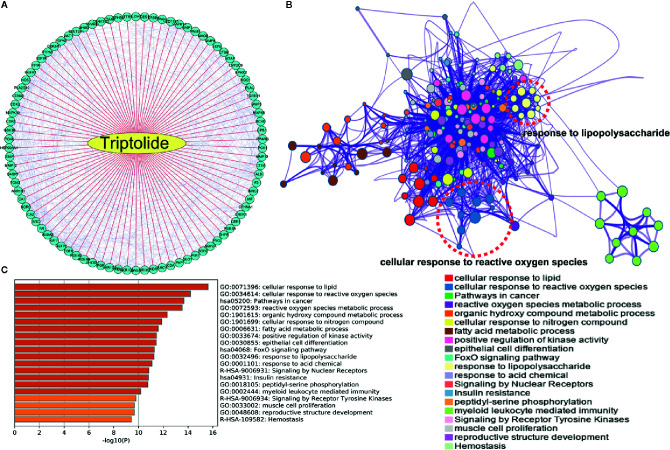
The underlying target of triptolide by network pharmacology analysis. **(A)** The potential targets of triptolide were analyzed by the PharmMapper website. The **(B)** PPI network and **(C)** heatmap showed the regulated top 10 KEGG and GO signaling pathways of triptolide by the Metascape website.

To explore the potential mechanism of triptolide, network pharmacology analysis at the PharmMapper website was used to identify the underlying molecule(s) regulated by triptolide. The potential targets of triptolide are shown in **Figure 4A**. To characterize the mechanism of triptolide, KEGG and GO analysis was utilized at the Metascape website. The PPI network and heatmap showed that the regulated signaling pathway of triptolide was involved in many physiological and pathological processes, including the cellular response to ROS and LPS (**Figures 4B, C**).

**Figure 5:**
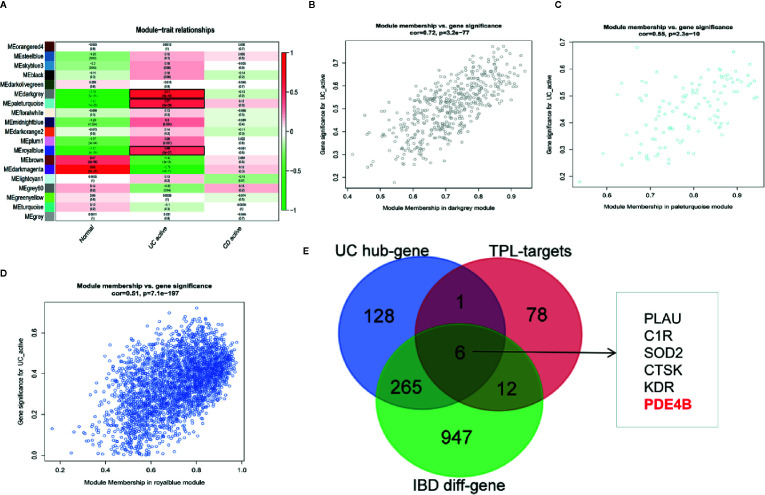
WGCNA was used to identify IBD-associated modules and hub genes in inflammatory bowel diseases. The co-expressive modules of IBD are depicted **(A)**. The correlation coefficient and p-value of **(B)** “dark gray”, **(C)** “pale turquoise”, **(D)** “royal blue” modules is shown **(B–D)**. **(E)** The Venn diagram shows the underlying molecules of triptolide in intestinal inflammation.

WGCNA can be used for finding co-expressive modules and hub genes. To explore the potential regulator(s) of IBD, WGCNA was used to identify IBD-associated hub genes. A total of 21,211 annotated-mRNA (74 UC-active samples, eight CD-active sample and 11 nontumor samples) were obtained from the GEO dataset (GSE75214). The analytical process is depicted in **Figure S4**. The results showed that the “dark gray”, “pale turquoise”, and “royal blue” modules were most closely related to UC-active samples (**Figures 5A–D**). In addition, 1,279 DEGs between UC-active samples and normal samples were selected by limma R package (log twofold change FC >1 and an adjusted P value <0.05) (**Figure S5** and **Table S3**). The intersected data of DEGs and hub genes from UC-active samples and the targets of triptolide indicated that PLUA, C1R, SOD2, CTSK, KDR and PDE4B may be regarded as potential therapeutic targets of triptolide in IBDs (**Figure 5E**).

### Bioinformatics Analysis Indicated the Underlying Biological Function of PDE4B in Inflammatory Bowel Diseases

**Figure 6:**
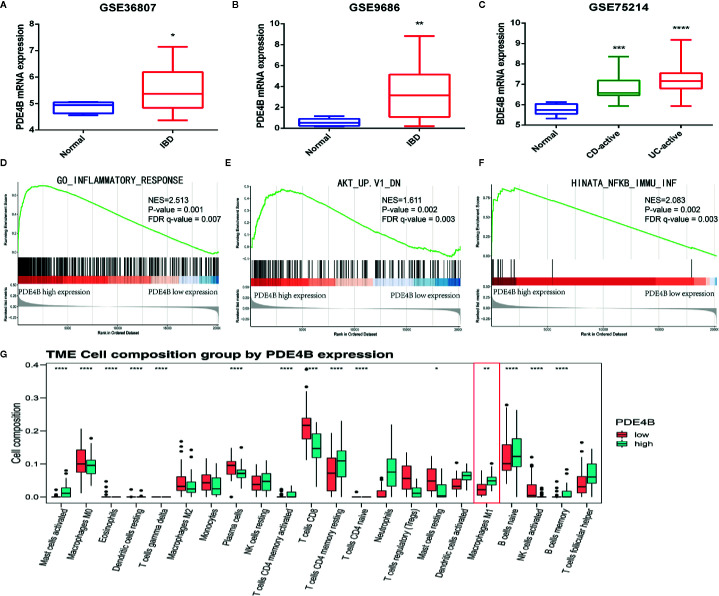
The biological function of PDE4B in inflammatory bowel diseases by bioinformatics analysis. **(A–C)** The box plot showed the level of PDE4B between normal samples and IBD samples in GSE36807 cohort **(A)**, GSE9686 cohort **(B)**, and GSE75214 cohort **(C)**. **(D–F)** GSEA showed that PDE4B played a positive role in “inflammatory response” **(D)**, as well as the “AKT” **(E)** and “NF-*κ*B” **(F)** signaling pathways. **(G)** The box plot showed the correction of PDE4B level, and the infiltration of 22 types of immune cells was determined by the CIBERSORT R package. Data are shown as the mean ± SD. *P < 0.05; **P   0.01; ***P < 0.001; ****P < 0.0001.

DEGs, GSEA, and CIBERSORT analyses of the GSE75214 cohort were performed to assess the biological function of PDE4B in IBDs. PDE4B was found to be significantly upregulated in IBD tissue compared with normal tissue (**Figures 6A–C**). GSEA was used to explore the regulatory signaling pathway of PDE4B in IBDs. Results show highly expressed PDE4B is enriched in the “inflammatory response” (NES = 2.513, P = 0.001) signaling pathway (**Figure 6D**). In addition, high expression of PDE4B is related to the “AKT” (NES = 1.611, P = 0.002) and “NF-*κ*B” (NES = 2.083, P = 0.002) signaling cascades (**Figures 6E, F**), indicating the up-regulation of PDE4B expression in IBD tissue may be related to the “AKT” and “NF-*κ*B” signaling pathways. CIBERSORT analysis was performed to characterize the role of PDE4B in immune cell infiltration (**Figure S6A**) and demonstrated that the level of PDE4B was positively regulated in immune cell infiltration, especially of macrophages (**Figure S6B**). The percentage of macrophage infiltration was clearly increased in the high PBE4B-expressing group (**Figure 6G**). The results suggest that PDE4B may be an important regulator of IBDs.

### Triptolide Treatment Inhibited PDE4B/AKT/NF-*κ*B Axis in DSS-Induced Mice Colitis and in LPS-Activated Macrophages

**Figure 7:**
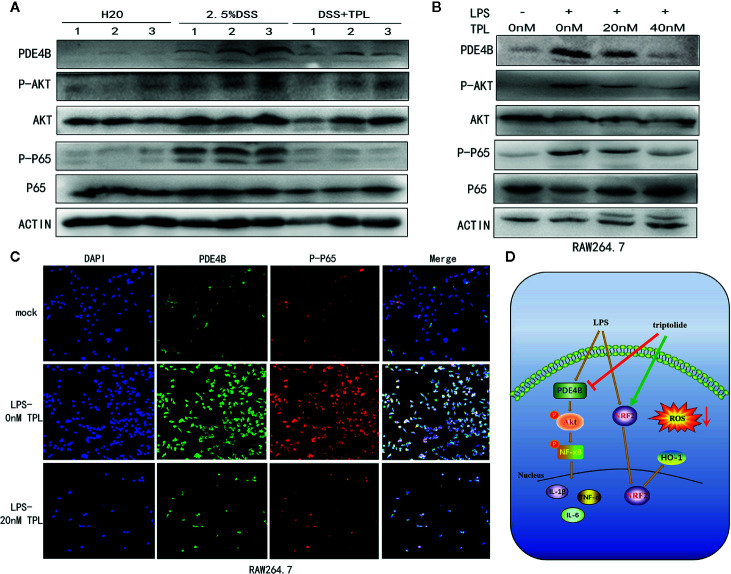
Triptolide treatment alleviated DSS-induced intestinal inflammation through the PDE4B/AKT/NF-*κ*B axis *in vivo* and *in vitro*. Mice aged 6–8 weeks were treated with 2.5% DSS with or without triptolide administration. **(A)** Western blots were used to measure the protein levels of PDE4B, total AKT, phosphorylated AKT, total P65, and phosphorylated P65 in colitis tissue. RAW 264.7 macrophages were treated with 1000 ng/ml LPS plus 0–40 nM triptolide for 24 h. **(B)** Western blots were used to measure the protein levels of PDE4B, total AKT, phosphorylated AKT, total P65, and phosphorylated P65 in RAW 264.7 macrophages. **(C)** Immunofluorescence assays were performed to determine PDE4B and phosphorylated P65 levels. **(D)** The flowchart of triptolide in the DSS-induced colitis model. Data are shown as the mean ± SD of at least three independent experiments.

To characterize the mechanism of action of triptolide on DSS-induced colitis in mice, western blotting was used to measure the regulatory proteins. The results showed that PDE4B was significantly upregulated in DSS-induced colitis tissue. However, triptolide treatment effectively inhibited this upregulation of PDE4B. In addition, the expression of phosphorylated AKT and phosphorylated P65 was also strongly suppressed by triptolide administration in DSS-induced colitis tissue (**Figure 7A**).

To further characterize the role of triptolide in the inflammatory process *in vitro*, RAW 264.7 cells were treated with 1,000 ng/ml LPS plus increasing amounts of triptolide for 24 h. Western blotting and immunofluorescence assays were used to characterize the altered genes. Our results showed that the level of PDE4B was significantly decreased by triptolide treatment. Consistently, triptolide treatment also effectively inhibited phosphorylated AKT and phosphorylated P65 expression (**Figure 7B**). In addition, using an immunofluorescence assay, we also found that PDE4B was dramatically inhibited by triptolide administration. Furthermore, triptolide treatment inhibited phosphorylated P65 expression in LPS-triggered macrophages (**Figure 7C**). Overall, the flowchart is shown in **Figure 7D**, and these results showed that triptolide treatment inhibited the PDE4B/AKT/NF-*κ*B signaling cascades in intestinal tract inflammation both *in vivo* and *in vitro*.

## Discussion

IBD is a group of chronic inflammatory diseases that affect the gastrointestinal tract and have a low mortality rate. However, the worldwide incidence of IBD is rising every year, putting a heavy burden on global health care systems (33). IBD is incurable, and current treatment strategies mainly include the use of anti-inflammatory steroids or immunosuppressants to reduce inflammation, dietary changes to try to remove environmental irritants, or surgery to remove damaged parts of the intestine to control the progression of IBD, but the efficacy of these strategies is not very satisfactory (34). The pathogenesis of IBD is characterized by dysregulation of the immune and inflammatory responses to unknown environmental triggers (35). Analyzing the mechanisms for immune imbalance and the inflammatory effects of IBD help us to better understand IBD and find new and effective therapeutic drugs or potential therapeutic targets. According to WGCNA, PDE4B was a potential hub gene of IBD, and PDE4B was also highly expressed in IBD tissue including active CD samples and active UC samples. GSEA indicated that PDE4B played a positive role in the “inflammatory response” signaling pathway, which closely correlated with IBD progression. In addition, PDE4B was also shown to be related to the level of immune cell infiltration (36, 37). Expression of PDE4B was positively correlated with the infiltration of M1 macrophages in our study, suggesting that PDE4B may promote the progression of inflammation. The above findings imply that PDE4B is a promising target in IBD since PDE4B activity is closely related to M1 type macrophage infiltration.

Triptolide, the main biologically active substance in *Tripterygium wilfordii* Hook F. (TwHF), was previously demonstrated to possess powerful anti-inflammatory and immunosuppressive properties in many preclinical studies (38, 39). Triptolide was found to regulate multiple inflammatory mediators *in vitro* and in animal models of inflammation and autoimmune diseases such as nephritis, asthma, and arthritis (40–42). Some studies also found that triptolide can relieve the intestinal inflammation of the chronic colitis observed in IL-10−/− mice (43, 44). However, the specific mechanism of action for triptolide in the regulation of intestinal inflammation has not yet been fully clarified. In this study, we found that intestinal inflammation of the DSS-induced mouse colitis model was significantly controlled and relieved after triptolide treatment, confirming that triptolide has a protective effect on intestinal inflammation. We identified the potential regulatory molecules of triptolide through pharmacological analysis, to investigate the potential mechanism of action of triptolide. Enrichment analysis of subsequent signaling pathways revealed that the regulatory factors of triptolide were mainly enriched in inflammation-related physiological and pathological processes, including the cellular responses to ROS and LPS, suggesting that triptolide is involved in the regulation of the inflammatory response. Our data suggested that triptolide treatment effectively inhibited DSS-induced colitis and that PDE4B may be a crucial regulatory molecule of triptolide.

It is well documented that macrophages play an important role in the regulation of the immune response and host defenses, and macrophages function as the primary regulators of inflammation and induced immune responses to endogenous and exogenous stimuli (45). Previous studies have determined that amounts of macrophages can be detected in colon samples from IBD patients and animal models, and they play an important role in the initiation and regression of inflammation (46, 47). It has also been reported that monocytes and proinflammatory M1 macrophages are significantly increased in the lamina propria of patients with IBD, which can destroy the epithelial barrier by relaxing tight junction proteins and inducing epithelial cell apoptosis, thereby driving the intestinal inflammation in IBD (14). The immunosuppressive effect of triptolide on macrophages has been confirmed in previous studies. Triptolide was found to inhibit the production of proinflammatory cytokines, inhibit the activity of inflammatory macrophages and eventually induce macrophage apoptosis (48–50). However, the specific mechanism of action of triptolide in regulating the function of macrophages is still unclear. In our study, in response to proinflammatory stimuli, induced macrophages undergo polarization to the M1 class and release large amounts of proinflammatory factors. Triptolide treatment significantly alleviated LPS-stimulated ROS production and proinflammatory cytokine expression, including those of IL-1β, IL-6, and TNF-α. In addition, triptolide treatment effectively suppressed macrophage differentiation in response to LPS and IFN*γ* stimuli and decreased the production of proinflammatory cytokines, which dramatically attenuated the LPS-activated inflammatory process in macrophages. ROS generation and elimination play important roles in many cellular processes, including the inflammatory response (51). Accumulating evidence shows that oxidative stress, characterized by excessive production of ROS, is closely related to the pathogenesis of IBD (52). During the onset of inflammation, neutrophils and macrophages infiltrate the intestinal mucosa at the site of IBD and release a large amount of ROS and cytokines, including IL-1β, IL-6 and TNF-α (53, 54). Excessive production of ROS will cause oxidative damage to DNA, protein and lipids, which will further promote the initiation and progression of IBD (55). In addition, LPS-activated macrophages disrupt the balance of intracellular oxidative stress levels and aggravate the inflammatory response (56). Targeting the site of inflammation and removing ROS may be an effective strategy to reduce IBD. Previous studies found that in the DSS-induced colitis mouse model, manganese Prussian blue nanozymes (MPBZs) with multiple enzymatic activities can accumulate in the inflamed area to inhibit the production of ROS, thereby reducing the intestinal inflammation (57). In this study, our results showed that triptolide treatment effectively decreased LPS-stimulated ROS generation in macrophages. Furthermore, triptolide treatment activated central antioxidant genes, such as the *NRF2/HO-1* signaling cascades in LPS-triggered macrophages. Triptolide treatment also suppressed downstream genes of the NRF2/HO-1 signaling pathway, including *GLCM, GSS*, and *MPO* in LPS-activated macrophages.

Further exploration of the specific molecular mechanisms of triptolide in inhibiting intestinal inflammation revealed that triptolide treatment inhibited the PDE4B/AKT/NF-*κ*B signaling cascades both *in vivo* and *in vitro*, which may be related to the effect of triptolide in relieving intestinal inflammation. PDE4B is a member of the type IV, cyclic AMP (cAMP)-specific, cyclic nucleotide phosphodiesterase (PDE) family and can hydrolyze cAMP and switch off cAMP signaling cascades (58). cAMP is considered to be an inducing agent for anti-inflammatory reactions, and the cAMP-dependent pathway has been widely used pharmacologically to treat inflammatory diseases (59). Previous studies have also revealed that PDE4B has a regulatory effect on the inflammatory process of the experimental model of lung injury (60). AKT (also known as protein kinase B, PKB) belongs to the AGC protein kinase subfamily. The overactivation of the PI3K/PKB axis in tumors substantially induces inflammatory tumor microenvironments (61). In addition, AKT promotes mitochondrial oxygen consumption and the accumulation of ROS by stimulating oxidative metabolism (62). The NF-*κ*B transcription factor was discovered 30 years ago and is composed of five subunits [RELA (p65), RELB, c-REL, p50, and p52] (63, 64). It is well known that NF-*κ*B is a critical regulator of the immunological response, inflammatory processes and ROS generation (65). PDE4B has been shown in previous studies to mediate the activity of AKT by regulating the hydrolysis of cAMP to influence its signal conduction (66, 67). Activated AKT is known to phosphorylate I*κ*K, a kinase of I*κ*B*α* (a direct inhibitor of NF-*κ*B), thus AKT can act as an upstream molecule of NF-*κ*B under acidic conditions (68). Our results indicated that PDE4B was significantly upregulated in IBD tissue and LPS-induced macrophages. Interestingly, PDE4B was clearly inhibited by triptolide administration in both DSS-induced colitis and LPS-activated macrophages. In addition, triptolide treatment effectively suppressed the level of phosphorylated AKT and P65 *in vitro* and *in vivo*. The results suggest that triptolide treatment to reduce DSS-induced colitis may be related to the inhibition of the PDE4B/AKT/P65 signal cascade.

In conclusion, we found that triptolide treatment relieves intestinal inflammation and protects the intestinal tract by inhibiting macrophage infiltration and M1 type polarization, reducing the expression of inflammation-related factors and the generation of ROS. Triptolide treatment significantly attenuated the inflammatory response of human monocytes and mouse macrophages and reduced the expression of proinflammatory cytokines and the production of ROS triggered by LPS. We also further elucidated the molecular mechanism of action of triptolide in alleviating the inflammatory response, which include inhibition of the generation of ROS and interfering with the polarization of macrophages by activating the NRF2/HO-1 signaling pathway. And the inhibition of PDE4B/AKT/NF-*κ*B signaling cascade by triptolide may be related to the effect of triptolide in alleviating intestinal inflammation. Our findings provide new ideas and options for IBD treatment.

## Data Availability Statement

The raw data supporting the conclusions of this article will be made available by the authors, without undue reservation.

## Ethics Statement

The animal study was reviewed and approved by Experimental Animal Welfare Ethics Review Committee of Zhejiang University.

## Author Contributions

JJ and ZZ conceived and designed the experiments. BT, JZ, and BZ performed the experiments. BZ, FW, YW, MC, and QW analyzed the data. SF, LZ, YY, RQ, and MX contributed reagents, materials, and analysis tools. BT and JZ wrote the paper. JJ edited the paper. All authors contributed to the article and approved the submitted version.

## Funding

This study was supported by the National Natural Science Foundation of China (Nos. 81803778, 81573657) and The Key Research and development Project of Zhejiang Province (No. 2018C0302) and The Medical and Health Care Key Project of Zhejiang Province (No. WKJ-ZJ-1629) and Natural Science Foundation of Zhejiang Province (No. LQ17H180001) and The Public Welfare Project of Zhejiang Province (Nos. 2016C37101, 2017C33216, and LGF18H160035).

## Conflict of Interest

The authors declare that the research was conducted in the absence of any commercial or financial relationships that could be construed as a potential conflict of interest.

## Supplementary Material

Supplementary Figure 1 The flowchart of triptolide treatment in the dextran sulfate sodium (DSS)-induced murine experimental colitis model.

Supplementary Figure 2 The model of DSS-induced murine experimental colitis with or without triptolide treatment.

Supplementary Figure 3 Triptolide treatment inhibited macrophage polarization in human macrophages. U937 cells were stimulated with 500 ng/ml LPS and 100 ng/ml IFN*γ* with or without 20 nM triptolide for 24 h. RT-qPCR was used to measure the levels of M1-mediated cytokines such as IL-1β **(A)**, IL-6 **(B)**, TNF-α **(C)**, and CXCL10 **(D)** and M1-mediated cytokines including Arg-1 **(E)** and CCL22 **(F)**. *β*-actin served as an internal control. Data are shown as the mean ± SD of at least three independent experiments. *P < 0.05; **P < 0.01; ***P < 0.001.

Supplementary Figure 4 The analytical process of WGCNA. **(A)** Sample dendrogram and trait heatmap in GSE75214. **(B)** Identification of the optimal soft threshold. **(C–E)** Construction and visualization of co-expression modules.

Supplementary Figure 5 Identification of DEGs between UC-active samples and normal samples in GSE75214 cohort. The volcano plot shows the DEGs between UC-active samples and normal samples (log twofold change FC >1 and an adjusted P value <0.05).

Supplementary Figure 6 Identification of immune cell infiltration in GSE75214 cohort. **(A)** The composition of immune cells in the tumor microenvironment in GSE75214 cohort. **(B)** The radar chart showed the correction of PDE4B level, and the infiltration of 22 types of immune cells was determined by the CIBERSORT R package.

Supplementary Table 1 Sequences of primers used for RT-PCR.

Supplementary Table 2 Antibodies used in the western blot and FACS.

Supplementary Table 3 Identification of DEGs between UC-active samples and normal samples.
