# Supplementary figures and images for: Therapeutic Potential of Triptolide as an Anti-Inflammatory Agent in Dextran Sulfate Sodium-Induced Murine Experimental Colitis
Source: Front Immunol. 2020 Nov 9;11:592084. doi: 10.3389/fimmu.2020.592084 (PMC7680904; doi:10.3389/fimmu.2020.592084)

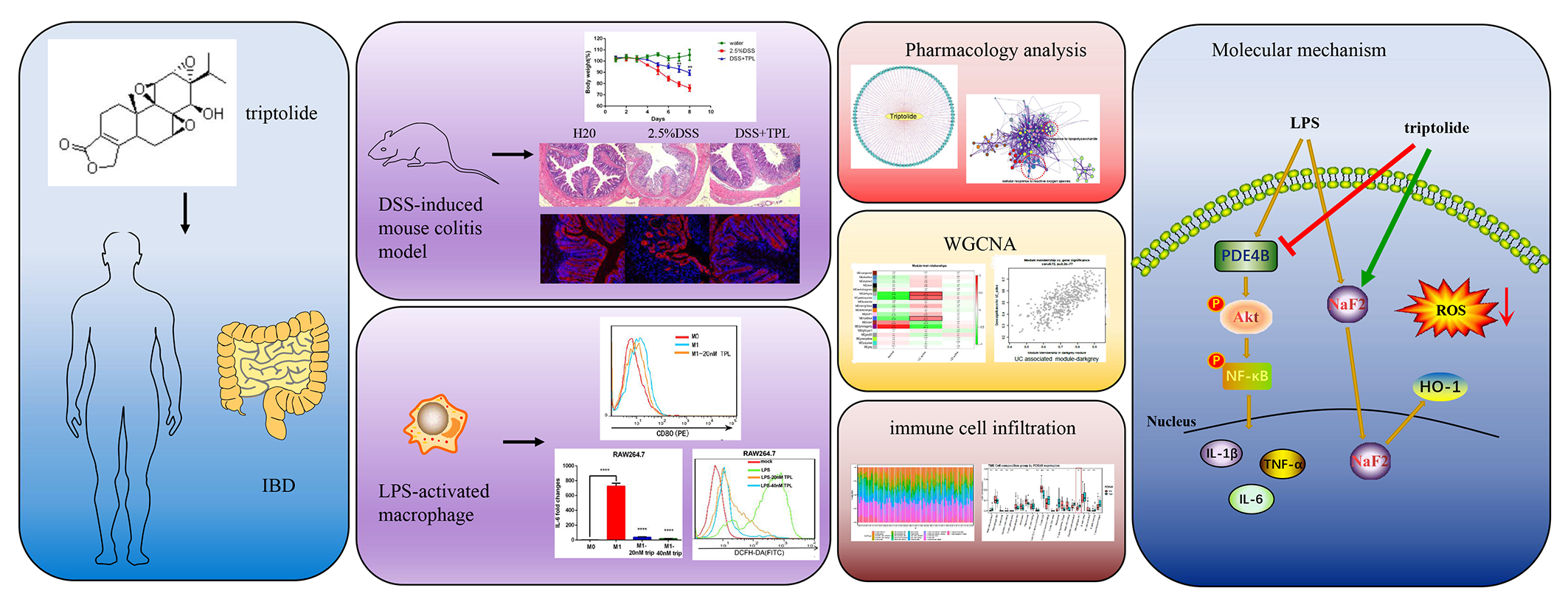

Supplement: Supplementary Figure 1 — The flowchart of triptolide treatment in the dextran sulfate sodium (DSS)-induced murine experimental colitis model. [file Image_1.tif]

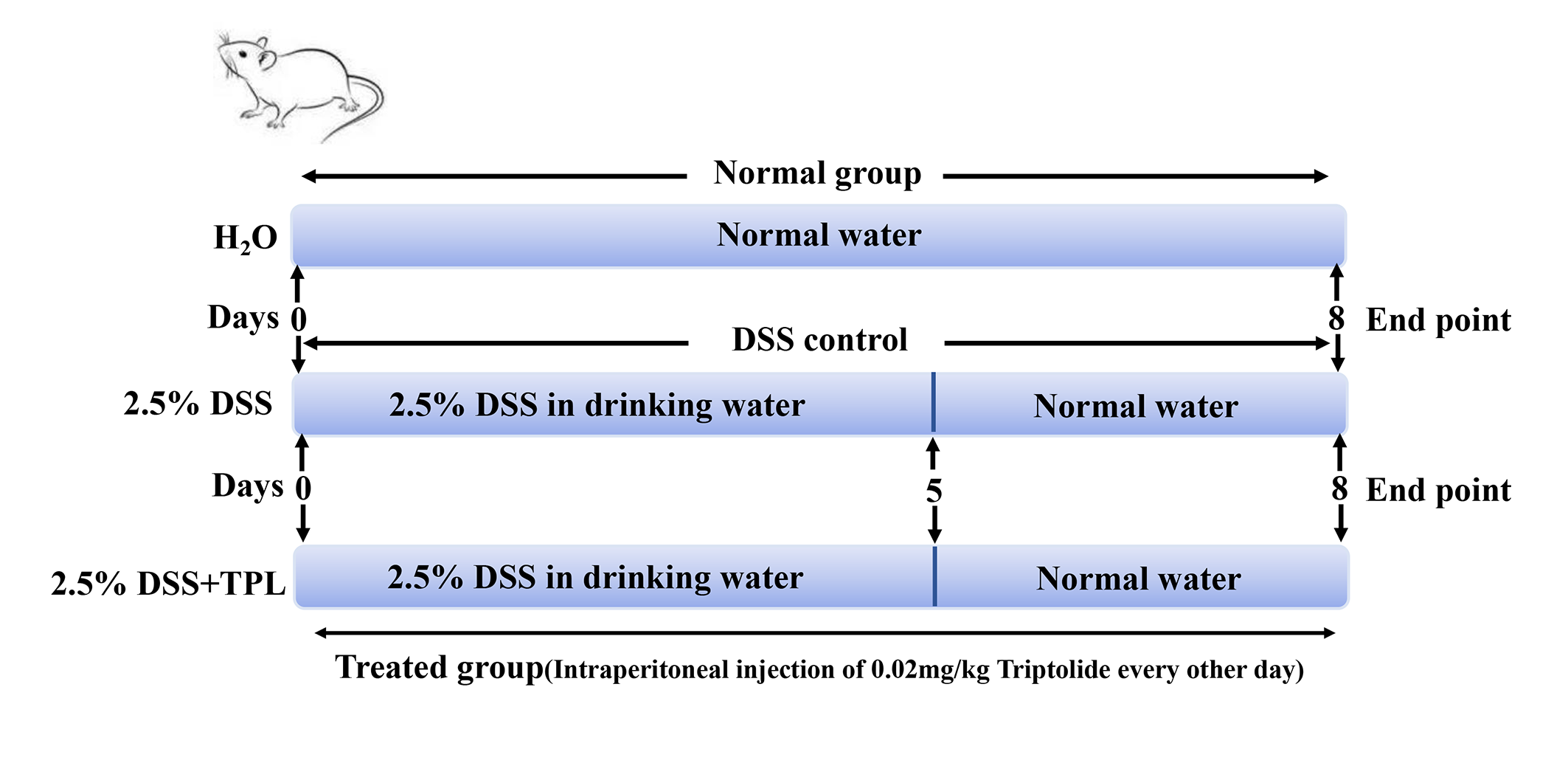

Supplement: Supplementary Figure 2 — The model of DSS-induced murine experimental colitis with or without triptolide treatment. [file Image_2.tif]

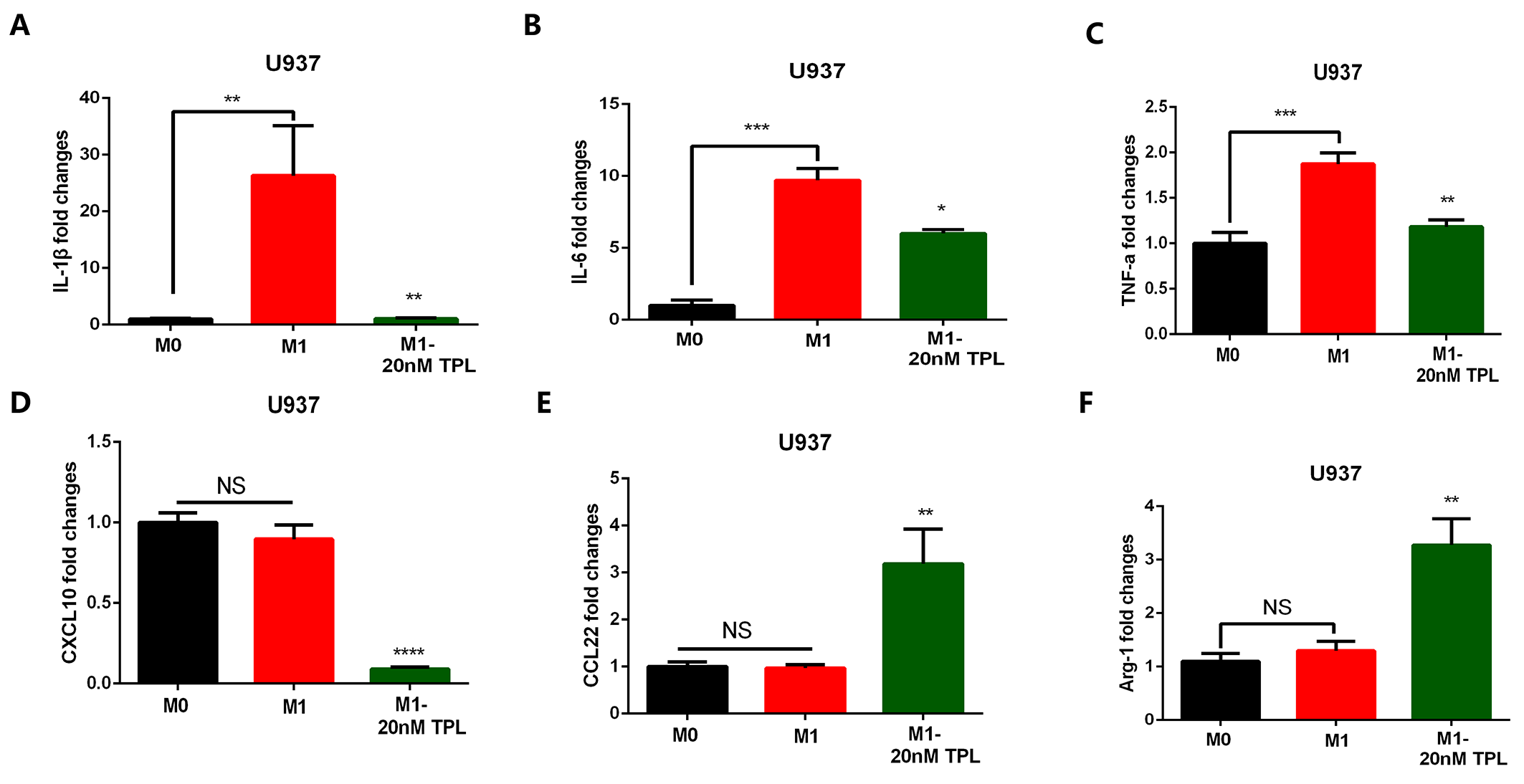

Supplement: Supplementary Figure 3 — Triptolide treatment inhibited macrophage polarization in human macrophages. U937 cells were stimulated with 500 ng/ml LPS and 100 ng/ml IFNγ with or without 20 nM triptolide for 24 h. RT-qPCR was used to measure the levels of M1-mediated cytokines such as IL-1β (A), IL-6 (B), TNF-α (C), and CXCL10 (D) and M1-mediated cytokines including Arg-1 (E) and CCL22 (F). β-actin served as an internal control. Data are shown as the mean ± SD of at least three independent experiments. *P < 0.05; **P < 0.01; ***P < 0.001. [file Image_3.tif]

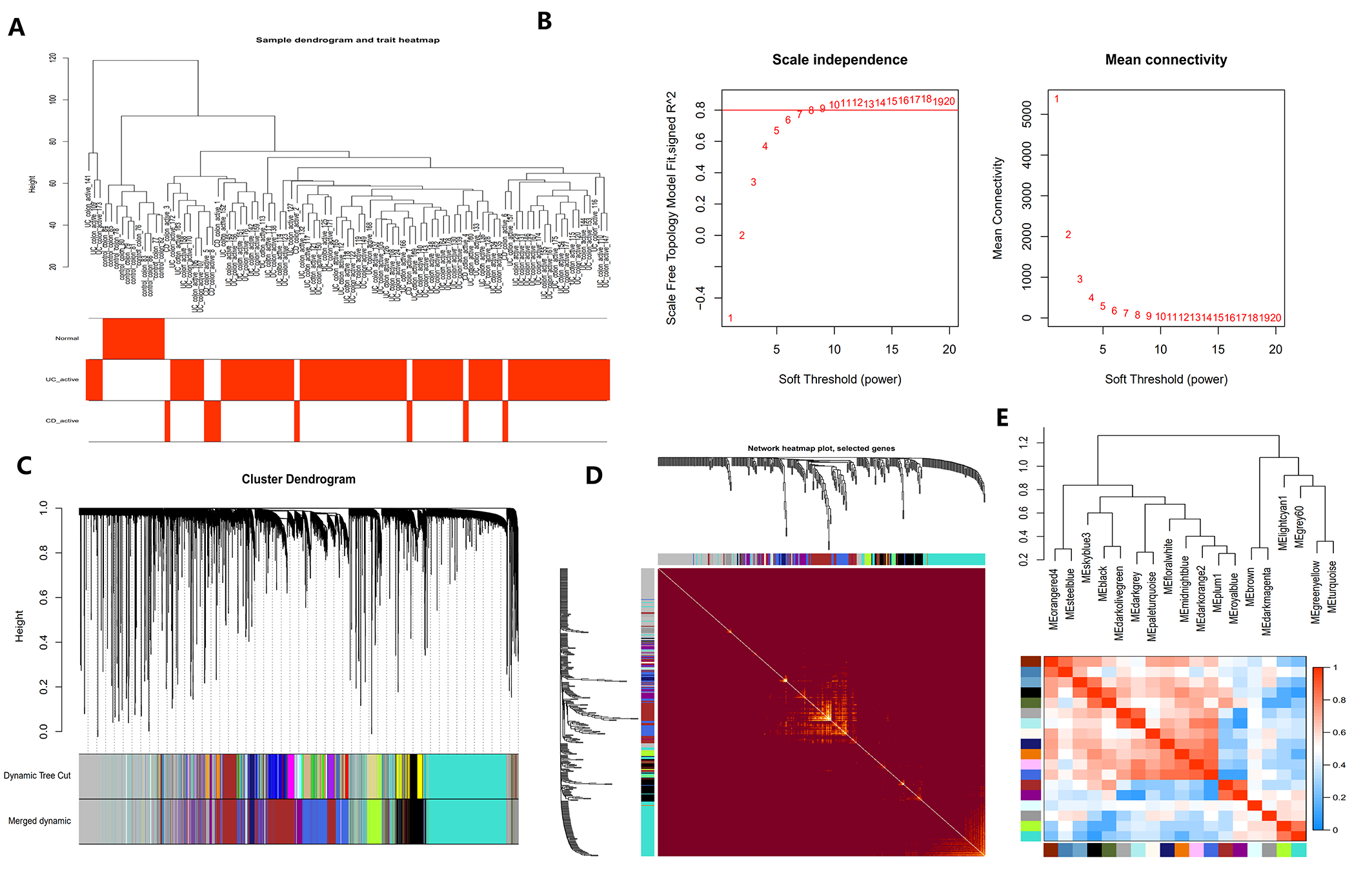

Supplement: Supplementary Figure 4 — The analytical process of WGCNA. (A) Sample dendrogram and trait heatmap in GSE75214. (B) Identification of the optimal soft threshold. (C–E) Construction and visualization of co-expression modules. [file Image_4.tif]

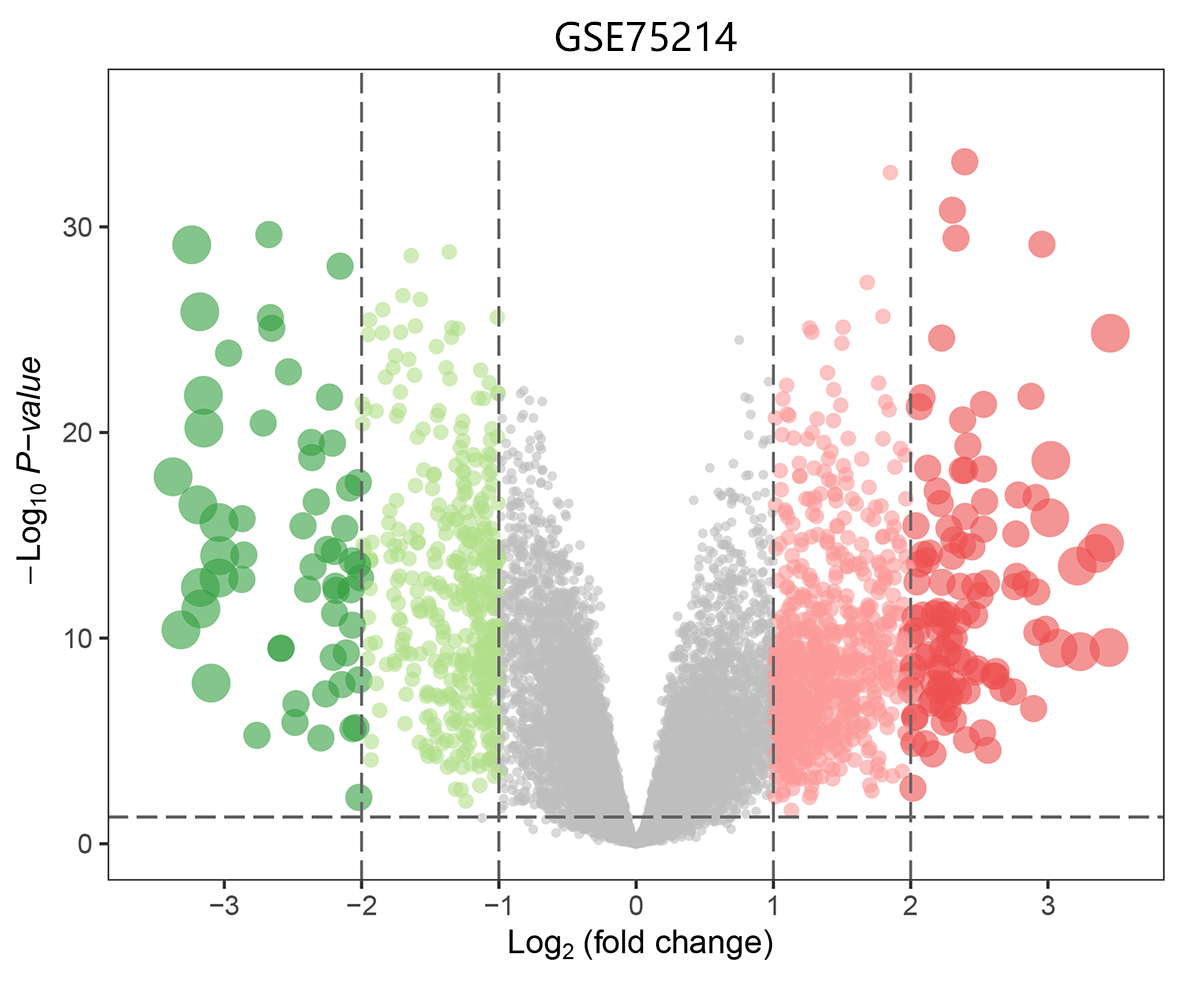

Supplement: Supplementary Figure 5 — Identification of DEGs between UC-active samples and normal samples in GSE75214 cohort. The volcano plot shows the DEGs between UC-active samples and normal samples (log twofold change FC >1 and an adjusted P value <0.05). [file Image_5.tif]

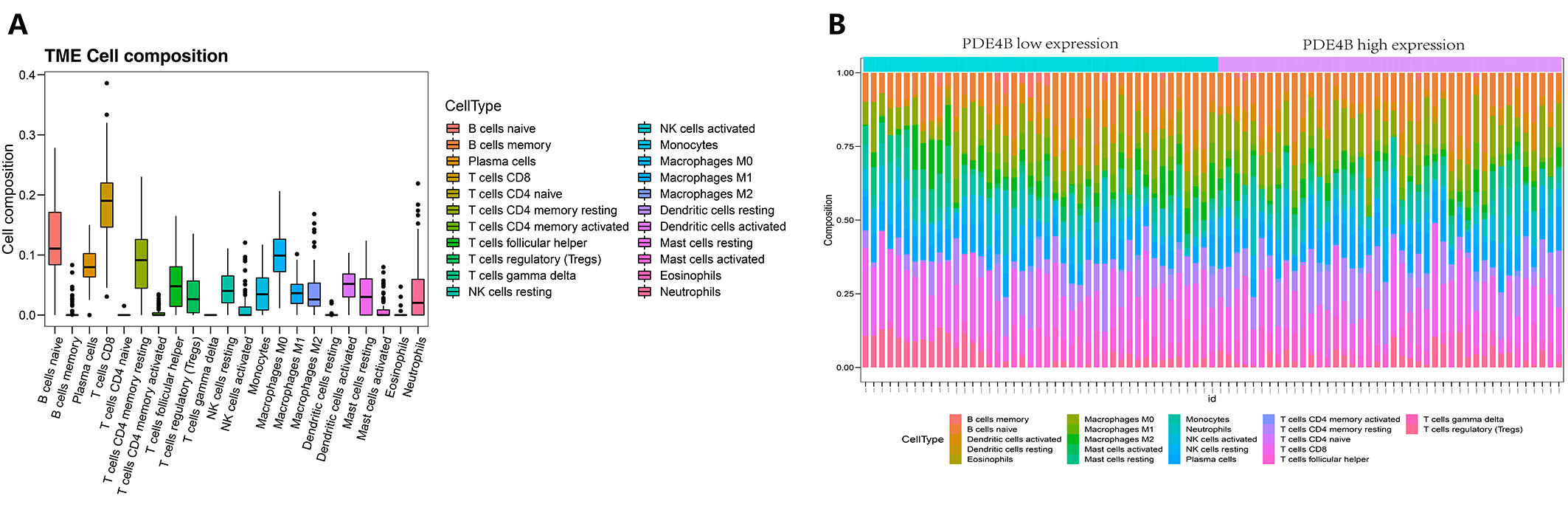

Supplement: Supplementary Figure 6 — Identification of immune cell infiltration in GSE75214 cohort. (A) The composition of immune cells in the tumor microenvironment in GSE75214 cohort. (B) The radar chart showed the correction of PDE4B level, and the infiltration of 22 types of immune cells was determined by the CIBERSORT R package. [file Image_6.tif]
